# Color Pattern on the Forewing of *Micropterix* (Lepidoptera: Micropterigidae): Insights into the Evolution of Wing Pattern and Wing Venation in Moths

**DOI:** 10.1371/journal.pone.0139972

**Published:** 2015-10-05

**Authors:** Sandra R. Schachat, Richard L. Brown

**Affiliations:** 1 Mississippi Entomological Museum, Mississippi State, Mississippi, United States of America; 2 Department of Paleobiology, Smithsonian Institution, Washington, District of Columbia, United States of America; CNRS, FRANCE

## Abstract

Wing patterns are key taxonomic characters that have long been used in descriptions of Lepidoptera; however, wing pattern homologies are not understood among different moth lineages. Here, we examine the relationship between wing venation and wing pattern in the genus *Micropterix*, among the most basal extant Lepidoptera, in order to evaluate the two existing predictive models that have the potential to establish wing pattern element homologies for the order. The location of wing pattern elements along the costal margin of the wing in *Micropterix* is consistent with the predictions of the model proposed for Tortricidae by Brown and Powell in 1991, later modified by Baixeras in 2002. The predictive power of this model for such distantly related taxa suggests that the model may hold across various superfamilies within Lepidoptera, and supports the long-held notion that fasciae, not spots, are the most likely primitive wing pattern elements for the order. In addition, the location of wing pattern elements suggests that the wing vein commonly termed Sc_1_ may in fact be a different vein, which Comstock identified in Trichoptera and referred to as “a.”

## Introduction

Many recent studies have examined the evolution of wing patterns in butterflies [1,2] and other macrolepidoptera [3–6]. The wing patterns of these taxa, and of other relatively derived moths such as Pyraloidea, are based on symmetry systems, which occur in different arrangements in various lineages [7] and consist of parallel lines in two or more colors overlaid on a light ground color [8–11]. Early-diverged moths, often small and brown, lack symmetry systems, and the evolutionary origin of their wing patterning is not known. Workers who study microlepidoptera have concluded that transverse bands, or fasciae, are primitive wing pattern elements for Lepidoptera and are constrained by venation [12–14], whereas workers who primarily study macrolepidoptera have concluded that spots, either “erratic” [7] or constrained by venation [15], are primitive. Both fasciae and spots can be found in basal Lepidoptera [7,14]. Largely due to the fact that these basal lineages are poorly studied, homologous wing pattern elements have not yet been established for the order.

The current lack of knowledge regarding wing pattern homology is of great concern because wing patterning has been used to describe and differentiate species throughout the history of Lepidoptera systematics. In recent years, great progress has been made in the application of molecular data toward the lepidopteran tree of life [16–18]. Because of the strong support for an integrated morphological and molecular approach to systematics [19,20], particularly for Lepidoptera [21–25], the use of wing patterns as taxonomic characters would supplement other morphological characters, e.g., genitalia and venation, and improve the resolution of the lepidopteran tree of life. Because genitalia and venation are skeletal elements, their different components are relatively easy to isolate. Wing pattern, in contrast, is repetitive and can change drastically with few or no changes to skeletal characters such as wing venation. However, the fact that wing pattern homologies are not understood prevents the use of this character in large-scale phylogenetic studies. Also due to the poor understanding of homology in this area, inconsistent terminology is used to describe wing pattern elements, especially between different families.

### Predictive Models

**Fig 1:**
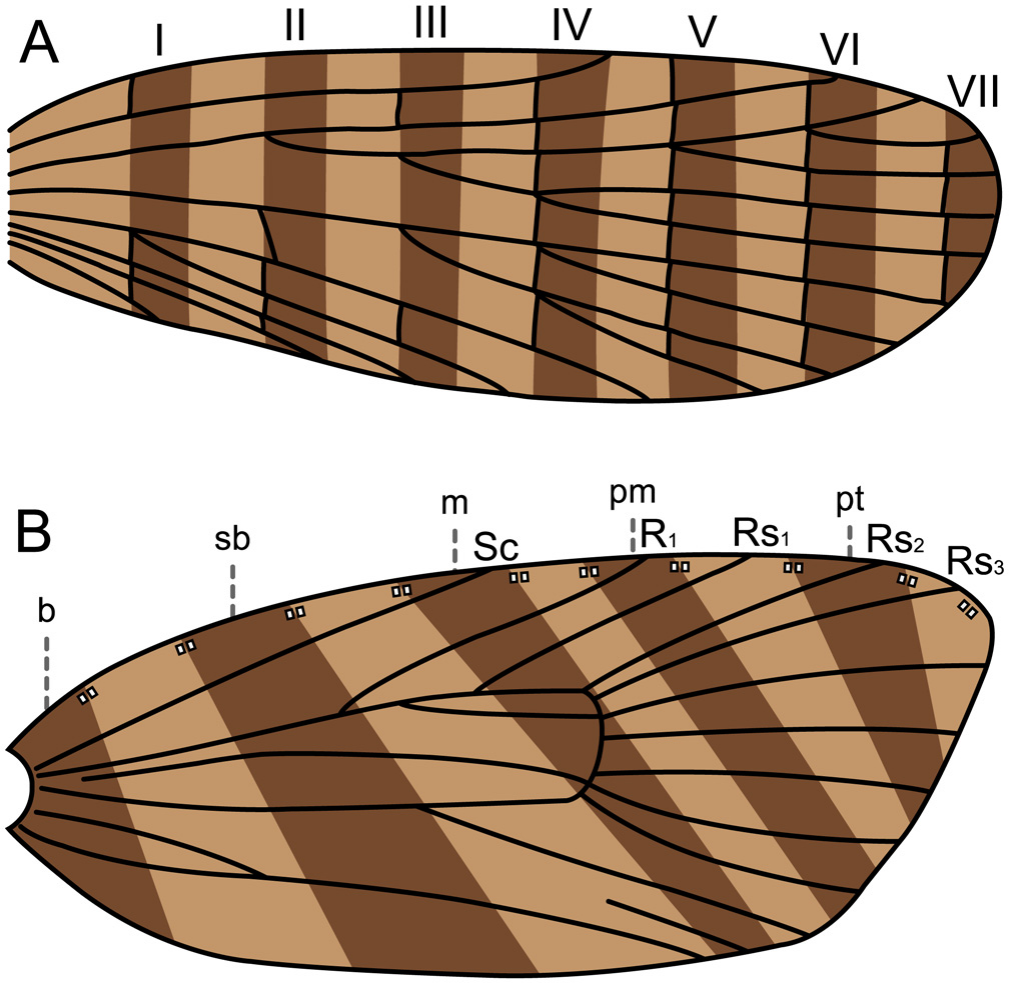
The two predictive models that relate wing pattern to wing venation. The beige areas of the wing represent the lighter, interfascial areas; the dark brown areas represent the fasciae. (A) The “vein-fork” model and associated nomenclature for fasciae (I-VII) shown on a panorpoid wing, following Lemche [14]. (B) The “wing-margin” model and associated nomenclature, following Baixeras [28]. Legend: **b**: basal; **sb**: subbasal; **m**: median; **pm**: postmedian; **pt**: preterminal.

Wing venation has long been suspected to constrain lepidopteran wing patterns [12,15,26]. Two models predict primitive forewing patterning for Lepidoptera (Fig 1); both assume that fasciae, not spots, are the primitive wing pattern elements. “Fasciae” are generally regarded to be transverse bands suffused with dark pigment, interspersed between interfascial areas that are suffused with the lighter pigment corresponding to the ground color. The first model [14,27], termed the “vein-fork” model here, posits that the basal edge of each fascia falls directly on the points where veins branch within the wing (Fig 1A). The second, termed the “wing-margin” model here, predicts the location of fasciae based on pairs of costal strigulae, or light markings, that always occur between the same veins along the costal margin of the wing in Tortricidae [13,28]. In the “wing-margin” model, fasciae are interspersed with interfascial areas between alternating pairs of costal strigulae, and so their location is thus constrained by the wing venation (Fig 1B). Therefore, the points where veins meet the wing costa constrain the location of fasciae, although some pairs of strigulae are not separated by veins on the tortricid wing; certain ancestral veins are not expressed in the adult wing. Various authors have explored the wing pattern structure in Tortricidae [29–31], but we emphasize the “wing-margin” model here because of its predictive potential. Both models are based on relatively derived moths: the “vein-fork” model was originally inspired by Pyralidae and Noctuidae and later was evaluated in taxa including other Lepidoptera and Paleodictyoptera [14,27], and the “wing-margin” model has only been proposed for Tortricidae [13,28], later adopted with further explanation by Gilligan et al. [32].

### Family Micropterigidae

Micropterigidae has been considered to be at the very base of the lepidopteran phylogeny since Meyrick [33], either by itself [16,34] or with Agathiphagidae as a sister group to the remaining Lepidoptera [17,35]. The relationship of the genus *Micropterix* to other Micropterigidae has been in flux. Certain workers have long suspected that *Micropterix* occurs at the base of the family-level phylogeny, distantly related to all other micropterigid genera [36,37]. A more recent molecular study has confirmed the monophyly of *Micropterix*, but recovered the genus within a larger clade [38]. The oldest possible *Micropterix* fossil dates to the Early-Late Cretaceous boundary, approximately 100 million years before the present [39–41]. The oldest definitive *Micropterix* fossils, belonging to the species *M*. *immensipalpa*, date to the Lutetian Stage of the Middle Eocene, approximately 48 to 41 million years before the present [39,40,42–44].

**Fig 2:**
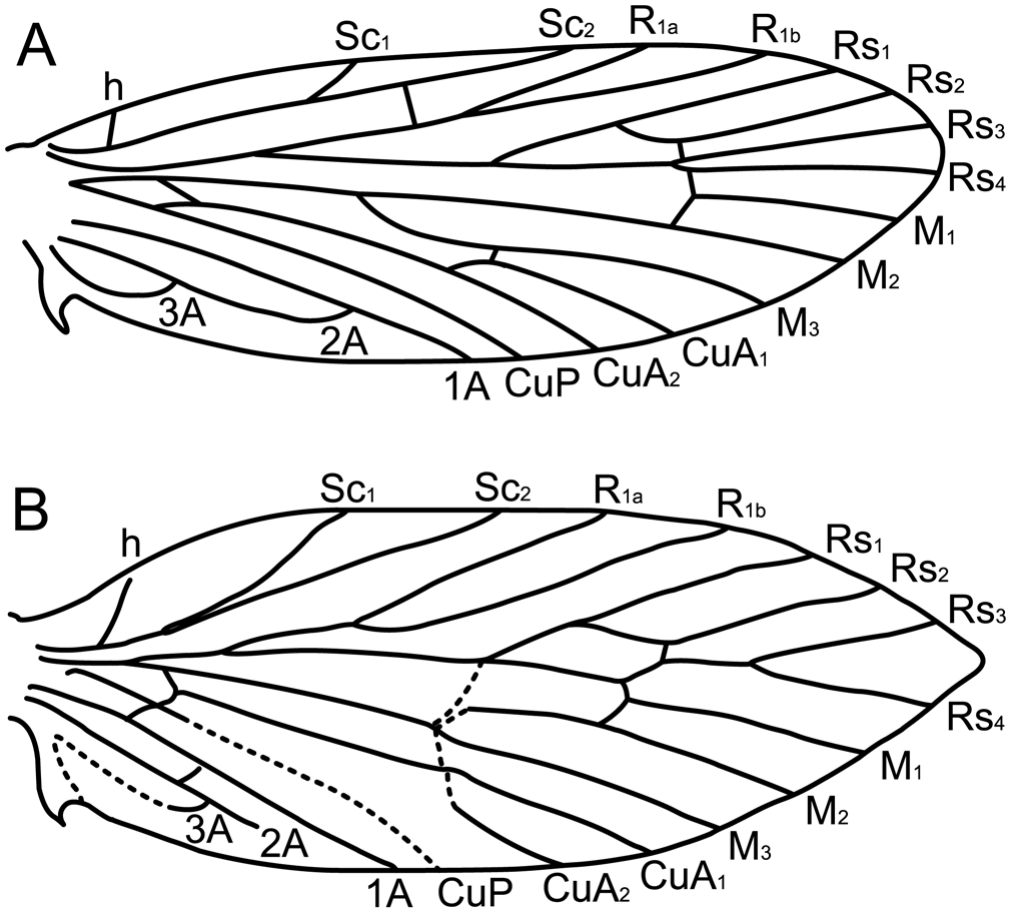
Primitive lepidopteran wing venation. (A) The wing venation groundplan for ancestral Lepidoptera [46]. (B) A micropterigid wing venation groundplan, based on the genus *Sabatinca* [47].

**Fig 3:**
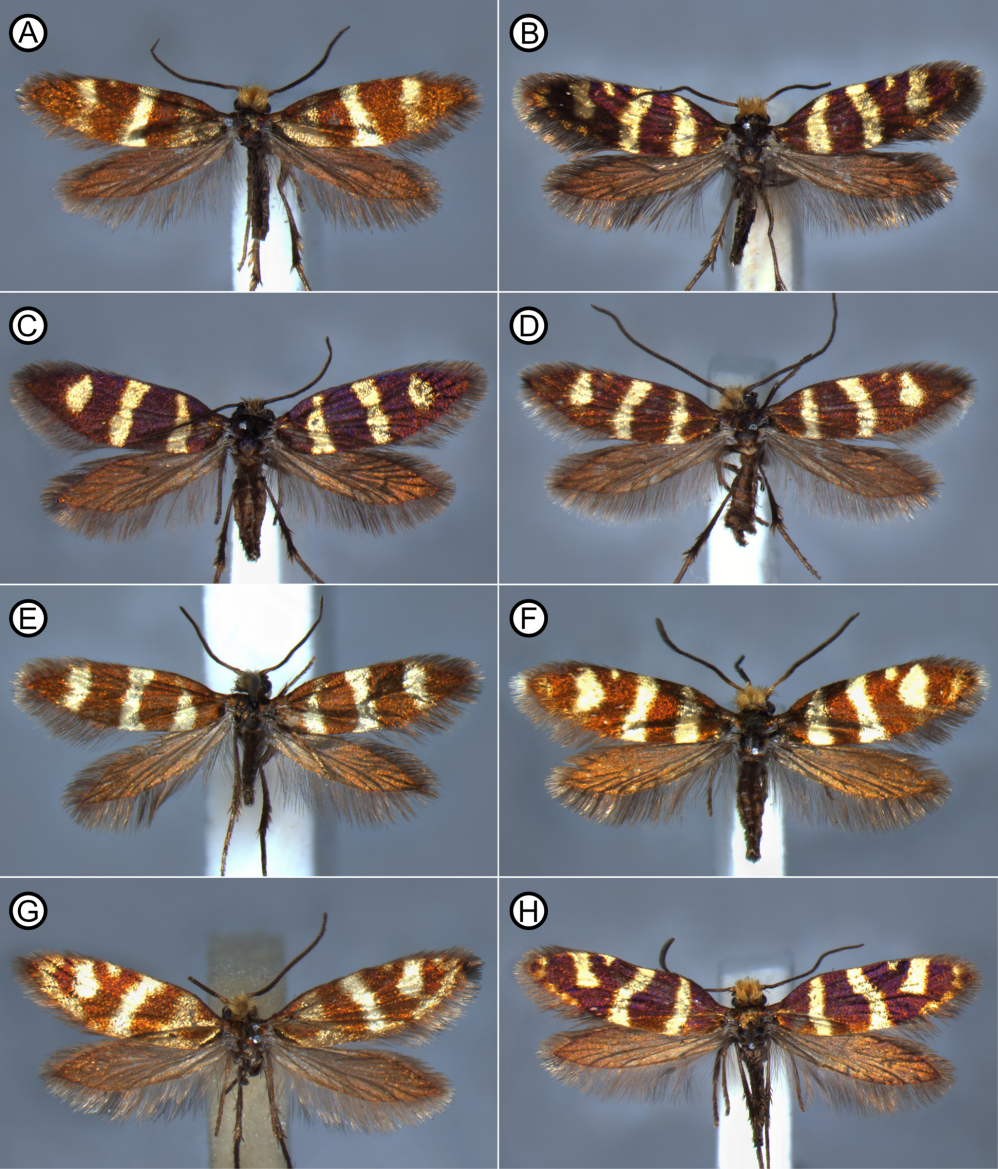
Photographs of some of the species examined in the present study, showing a sampling of the variety of *Micropterix* wing patterns. (A) *M*. *aglaella*. (B) *M*. *allionella*. (C) *M*. *aureatella*. (D) *M*. *aureatella*. (E) *M*. *rablensis*. (F) *M*. *rothenbachii*. (G) *M*. *schaefferi* 1. (H) *M*. *schaefferi* 2.

**Fig 4:**
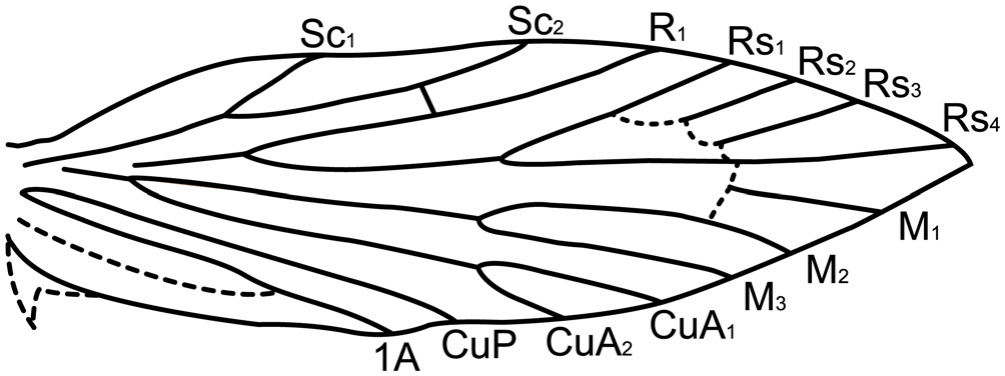
Wing venation of *Micropterix schaefferi* 2 labeled according to currently recognized nomenclature.

Micropterigid wing venation resembles the groundplan reconstructed for the common ancestor of all Lepidoptera (Fig 2). The present study focuses on *Micropterix* in particular because its forewing patterns consist exclusively of dark fasciae and light interfascial areas (Fig 3), whereas both fasciate and non-fasciate patterns are present in other micropterigid genera such as *Sabatinca*. Because wing patterns in *Micropterix* include only two colors, a light tan and dark purplish-brown, the distinction between fasciae and interfascial areas is straightforward and unambiguous. Wing patterns of *Micropterix* can vary among individuals of the same species, as well as between species [45], but there is little variation in wing venation (Fig 4). The varied forewing color patterns in this genus are therefore a suitable living analog for the primitive fasciate wing patterns in ancestral Lepidoptera assumed by both models discussed here.

## Materials and Methods

All of the specimens examined for this study are held in the USNM Entomology collections in Washington, DC, USA. A total of 12 species of *Micropterix* were examined. Recently, *M*. *anderschella* was found to be a synonym of *M*. *schaefferi* [45]. We examined specimens formerly assigned to both *anderschella* and *schaefferi*, and because we found different amounts of variation in these two groups, we decided to retain separate categories. Specimens that already belonged to *schaefferi* before 2007 are here called as “*schaefferi* 1,” and specimens that formerly belonged to *anderschella* are referred to as “*schaefferi* 2.” For each species included in the study, all available specimens were reviewed to determine the number of differentiated fasciae (fasciae separated by lightly colored interfascial areas) and the number of confluent fasciae, also called color fields (fasciae that cannot be differentiated due to suffusion of an interfascial area with dark scales). For species in which all individuals have wing patterns with the same number of differentiated fasciae, the forewing of one individual was illustrated. When forewing patterns with varying numbers of differentiated fasciae were observed among individuals of the same species, the venation-pattern relationship was recorded and illustrated for one representative of each variation. Likewise, variants with suffused interfascial areas and lack of expression in fasciae at the costa were illustrated.

Scaled wings, instead of cleared wings, were examined in order to observe the precise relationship between wing pattern and venation. Micropterigid wings are thinly scaled, and the venation becomes visible when specimens are lit from below using a microscope stage light. The observed wing venation was confirmed by examination of a wing slide of *M*. *schaefferi* 2 (USNM 91791) and the published literature [48]. To verify that the illustrations fully represent the species to which they correspond, up to 10 specimens per species—for a total of up to 20 forewings—were examined under a light microscope. (Results are discussed primarily in terms of wings instead of specimens because, in a few cases, only one forewing could be examined per specimen due to wear or due to the angle at which the specimen had been pinned. Furthermore, a number of specimens have pattern arrangements that vary between the two forewings.) To create illustrations, one forewing was photographed while backlit so that both the patterning and venation were visible. This photograph was used as a template for the wing venation/wing patterning schematic. The location of the wing vein 1A+2A could not be observed in all pinned specimens because of the overlap between the forewing and hindwing, and therefore had to be inferred based on previously described venation [48]; however, this vein is of no relevance to either model because it does not bifurcate in the sense of Lemche’s model, nor does it reach the costal margin. Similarly, the outline of the jugal lobe had to be inferred because this feature was often folded in the specimens examined; its outline was inferred based on previous descriptions [48]. Inferred features are illustrated with dashed lines.

We assessed support for each of the two models by determining whether the expected relationships between relevant characters were observed in *Micropterix*. The “vein-fork” model predicts that the basal edges of fasciae should lie along the points where veins bifurcate (Fig 1A); the points where veins meet the costal margin (costa) and inner margin (dorsum) should be unrelated to fasciae positions. In contrast, the “wing-margin” model predicts fasciae should meet the costa between the same veins as observed in Tortricidae (Fig 1B); under this model, the positions of the fasciae should be independent of the locations where wing veins bifurcate.

Süffert identified five basic pattern elements on lepidopteran wings: ripple patterns, dependent patterns (encompassing all pattern elements that depend on wing venation), eyespots (ocelli), crossbands (fasciae), and color fields [49]. These terms are in continuous use [13,50]. Because none of the *Micropterix* wing patterns studied were found to contain ripple patterns or eyespots and because the aim of the present investigation is to determine whether dependent patterns exist in *Micropterix*, the main terms used here are “fasciae” and “color fields.” Differentiated fasciae are transverse bands that are bordered by interfascial areas on each side. “Dark color” refers to the dark purple/brown color associated with fasciae. “Ground color” refers to the light beige color associated with interfascial areas. Because this term is conventionally used, it is employed here for the sake of continuity; however, we caution that “ground color” is not meant to imply any sort of priority or developmental sequence, nor should this imply lack of pigmentation. The apparent boundary between a fascia and an interfacial area can change in a number of ways. “Color fields” are wider patches that are formed when an interfascial area is subject to “complete suffusion”–the interfascial area is suffused entirely with dark color, so that the two adjacent fasciae appear “confluent.” A plus sign (+) is used here to denote the confluent fasciae embedded in a single color field. “Incomplete suffusion” refers to instances in which an interfascial area is partially suffused with dark color at the costal margin of the wing such that dark scales surround the vein that the interfascial area straddles; thus, the fascia appears to have expanded. “Incomplete lack of expression” refers to instances in which a fascia is not fully expressed at the costal margin of the wing so that ground color surrounds the vein that the fascia normally straddles; the interfascial area therefore appears to have expanded.

## Results

**Fig 5:**
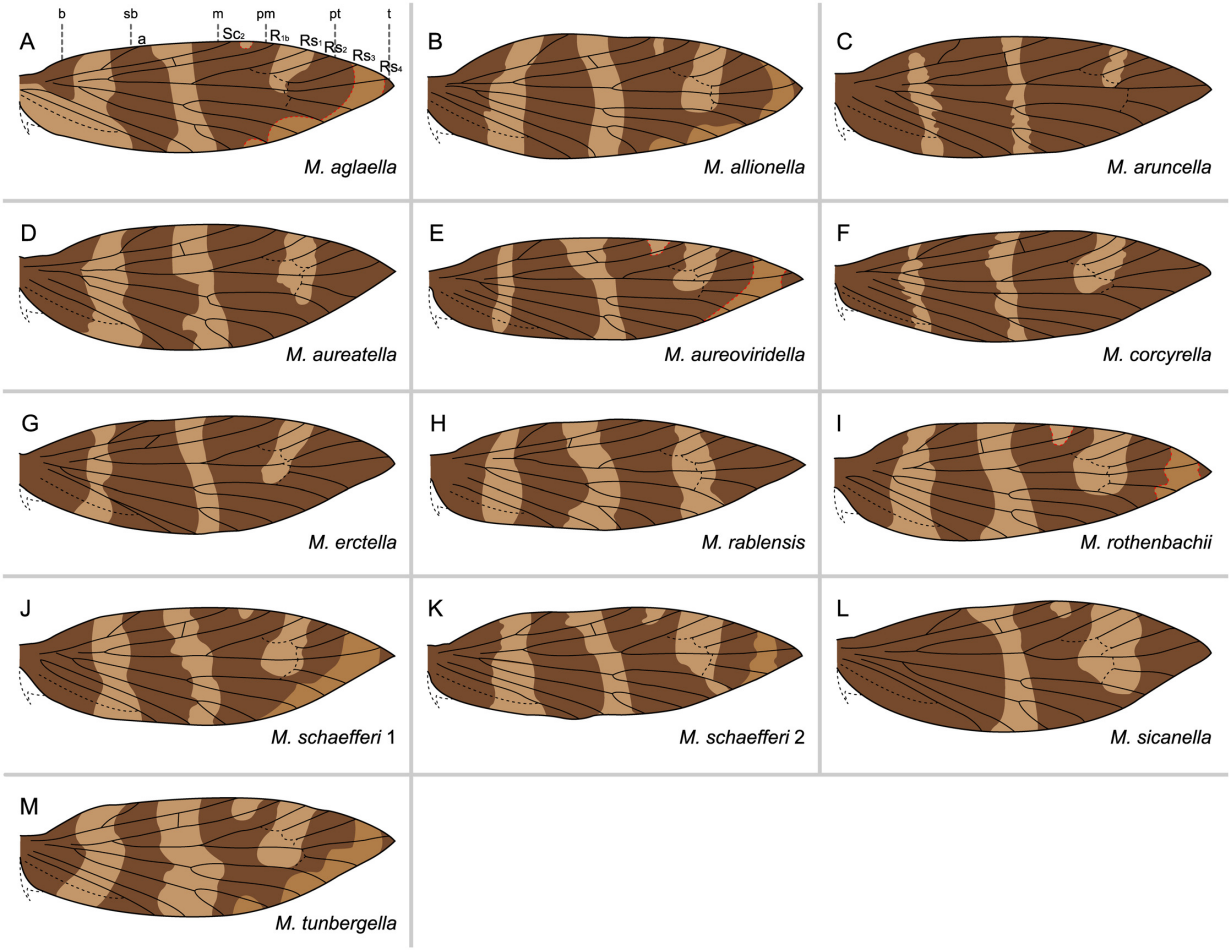
Wing patterns of *Micropterix* with no incomplete suffusion or lack of expression at the costa. For each species, dashed red lines indicate interfascial areas that are suffused with dark scales in some, but not all, specimens. For an explanation of the nomenclature used for veins on the wing costa, see Discussion. Legend: **b**: basal; **sb**: subbasal; **m**: median; **pm**: postmedian; **pt**: preterminal; **t**: terminal.

We examined a total of 172 forewings representing 12 species of *Micropterix* (Table 1). The species *M*. *aglaella*, *M*. *allionella*, *M*. *aureoviridella*, *M*. *rothenbachii*, *M*. *schaefferi* 1, *M*. *schaefferi* 2, and *M*. *tunbergella* have wing patterns with six differentiated fasciae, all separated by visible interfascial areas (Fig 5). Very few forks in the venation lie along or immediately adjacent to the basal edge of any fasciae. The basal fascia extends from the costa to the dorsum in all species except *M*. *aglaella*, in which this fascia extends from costa to midwing. The subbasal fascia extends from Sc_1_ on the costa to the dorsum. The median fascia extends from Sc_2_ on the costa down to the dorsum. The postmedian fascia originates from R_1_ on the costa but becomes confluent with the median fascia at midwing, with varying degrees of ground color between the two fasciae. The preterminal fascia originates from Rs_2_ and extends to M_2_ on the dorsum, usually becoming confluent with the median + postmedian fasciae near the dorsum. The terminal fascia is a spot of varying size at Rs_4_. The interfascial area that separates the terminal and preterminal fasciae is difficult to see under some types of lighting, and may be imperceptible in specimens that are old or worn. All fasciae are separated by interfascial areas, with the two most apical interfascial areas straddling or abutting Rs_1_ and Rs_3_, respectively, at the costa. The positions of these fasciae on the costa relative to venation are the same as those in wing pattern model proposed for Tortricidae [13,28] except that Tortricidae have only one Sc vein, and Rs_4_ intercepts the wing margin at the termen (rather than the costa in Micropterigidae) resulting in absence of a distinct terminal fascia. When present in tortricids, the remnant of the terminal fascia is sometimes termed an “apical spot.”

**Table 1:** Numbers of differentiated fasciae and color fields on the forewings examined for this study. For an explanation of terms used, see the last paragraph of the Methods.

Species	6 differentiated fasciae; 0 color fields	4 differentiated fasciae; 1 color field	2 differentiated fasciae; 2 color fields	Total
*M*. *aglaella*	1		19	20
*M*. *allionella*	6			6
*M*. *aruncella*			20	20
*M*. *aureatella*			20	20
*M*. *aureoviridella*	2		2	4
*M*. *corcyrella*			10	10
*M*. *erctella*			2	2
*M*. *rablensis*			20	20
*M*. *rothenbachii*	15	3	2	20
*M*. *schaefferi* 1	6			6
*M*. *schaefferi* 2	20			20
*M*. *sicanella*			4	4
*M*. *tunbergella*	20			20
**TOTAL**	**70**	**3**	**99**	**172**

Three of the *M*. *rothenbachii* wings examined have four differentiated fasciae, plus one color field produced by confluence of the preterminal + terminal fasciae (Fig 5I). The majority of species examined (9) include individuals whose wings have two color fields. Consequently, the forewing has four dark areas, only two of which are comprised of differentiated fasciae. *M*. *sicanella* has a unique wing pattern due to the interfascial areas that have become suffused: its two color fields are formed by confluent basal + subbasal and preterminal + terminal fasciae (Fig 5L). All examined specimens of *M*. *aruncella*, *M*. *aureatella*, *M*. *corcyrella*, *M*. *erectella*, and *M*. *rablensis* and 19 of 20 *M*. *aglaella* wings have color fields formed by suffusion of the interfascial areas between the median + postmedian and preterminal + terminal fasciae (Fig 5). The same pattern of suffusion of these interfascial areas can be seen in certain individuals belonging to *M*. *aureoviridella* and *M*. *rothenbachii*, two species that also include other specimens with six differentiated fasciae at the costa (Fig 5E and 5I). In these species with both differentiated fasciae and color fields, as in the other species discussed above, very few forks in the venation lie along the basal edge of a fascia.

**Fig 6:**
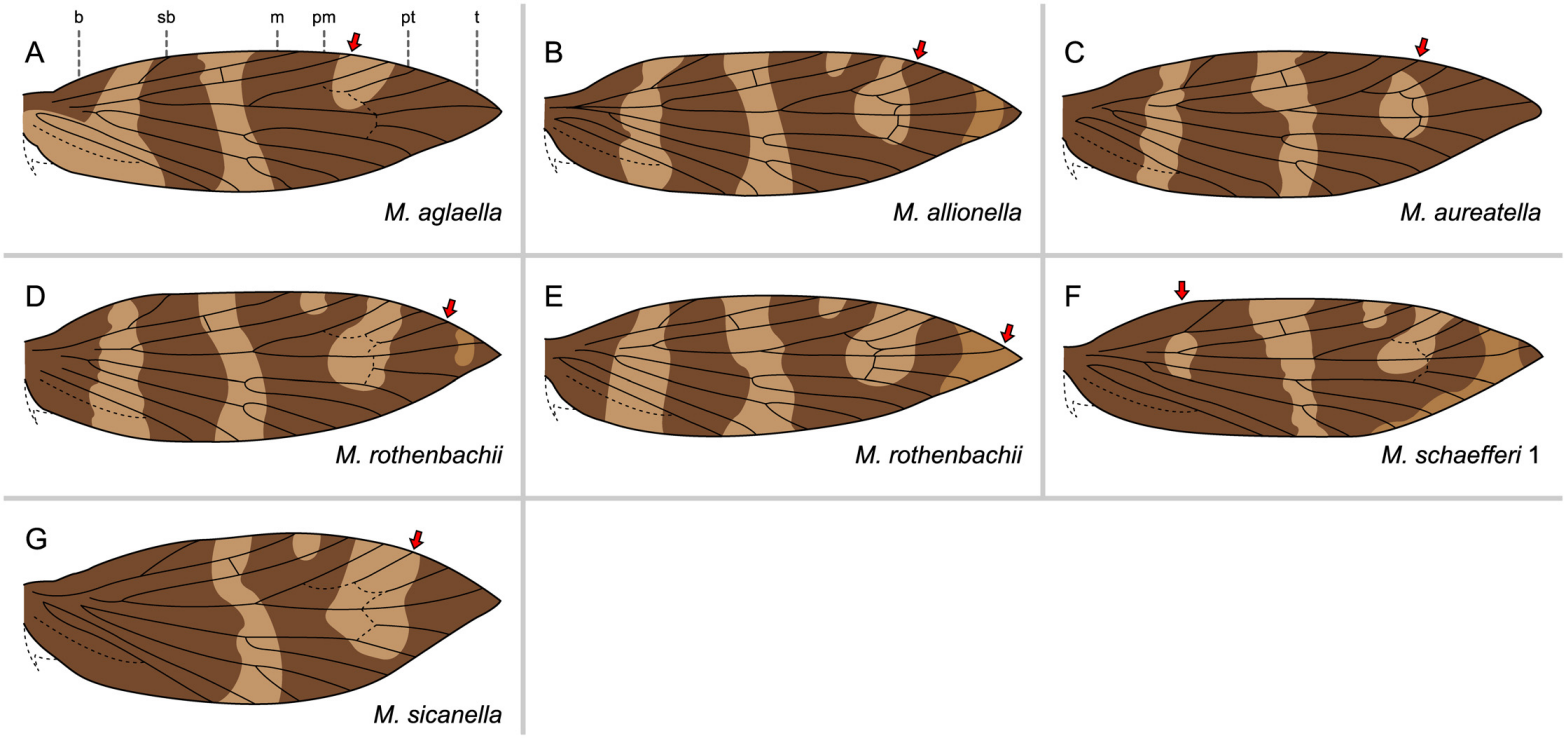
Wing patterns of *Micropterix* with variations of the groundplan at the costal margin of the wing, involving incomplete suffusion of interfascial areas of incomplete lack of expression of fasciae. The red arrows point to areas of incomplete suffusion/incomplete lack of expression at the wing costa. Legend: **b**: basal; **sb**: subbasal; **m**: median; **pm**: postmedian; **pt**: preterminal; **t**: terminal.

We found eight of the species examined (*M*. *aglaella*, *M*. *allionella*, *M*. *aruncella*, *M*. *aureatella*, *M*. *rablensis*, *M*. *rothenbachii*, *M*. *schaefferi* 1, *M*. *sicanella*) to contain individuals displaying incomplete suffusion of interfascial areas and/or lack of expression of fasciae at the wing costa (Table 2). In most cases, this involves the interfascial area between the postmedian + preterminal fasciae. One type of incomplete suffusion appears in *M*. *allionella*, *M*. *aglaella*, *M*. *aruncella*, *M*. *aureatella*, *M*. *rablensis*, *M*. *rothenbachii*, and *M*. *sicanella*: the interfascial area is suffused with dark color along the edge of the preterminal fascia, forming a larger dark pattern element that also straddles the Rs_1_ vein at the costa; this leaves a smaller patch of ground color that does not straddle or abut any vein at the wing costa (Fig 6B). On the wings of some *M*. *aureatella* specimens, the postmedian + preterminal fasciae are entirely confluent along the costa (Fig 6C); this may represent a further step in the suffusion process than that seen in Fig 6B. Suffusion of the basal-subbasal interfascial area is incomplete in *M*. *schaefferi* 1 (Fig 6F), and this is interpreted as an intermediate step between separate and confluent basal + subbasal fasciae.

**Table 2:** Total number of wings examined, and number of wings displaying incomplete suffusion or incomplete lack of expression at the wing costa, per species. For an explanation of terms used, see the last paragraph of the Methods.

Species	Total	Incomplete … at costa
*M*. *aglaella*	20	9
*M*. *allionella*	6	2
*M*. *aruncella*	20	6
*M*. *aureatella*	20	15
*M*. *aureoviridella*	4	
*M*. *corcyrella*	10	
*M*. *erctella*	2	
*M*. *rablensis*	20	6
*M*. *rothenbachii*	20	14
*M*. *schaefferi* 1	6	3
*M*. *schaefferi* 2	20	
*M*. *sicanella*	4	2
*M*. *tunbergella*	20	
**TOTAL**	**172**	**57**

Some specimens have one wing that follows the basic groundplan and one that shows incomplete suffusion or lack of expression at the costa. One *M*. *aglaella* specimen has incomplete lack of expression of the postmedian fascia such that the adjacent interfascial area appears to straddle not only Rs_1_ but also R_1_ at the costa (Fig 6A); this type of lack of expression is also present on one wing of a *M*. *rablensis* specimen whose other wing shows no modifications of the basic groundplan. Due to lack of expression of the preterminal fascia at the costa, this same interfascial area appears to have expanded in the opposite direction on the wings of two other *M*. *rablensis* specimens, and one wing of a *M*. *sicanella* specimen: a patch of ground color straddles Rs_1_ and also Rs_2_ at the costa (Fig 6G).

A few other modifications of the groundplan due to incomplete suffusion/lack of expression occur on other areas of the costa. In *M*. *schaefferi* 1, the basal + subbasal fasciae are confluent along the costa, but not at midwing (Fig 6F); this may be an intermediate step in the evolution of the wing pattern seen in *M*. *sicanella*, in which these fasciae are completely confluent (Fig 5L). Lastly, *M*. *rothenbachii* shows two modifications of the groundplan involving the interfascial area that precedes the terminal fascia. In the first, there is incomplete suffusion of this interfascial area along the boundary with the preterminal fascia at the costa (Fig 6D); this may represent an intermediate step in the evolution of the wing pattern with complete suffusion that was observed in other *M*. *rothenbachii* specimens (Fig 5I). Another *M*. *rothenbachii* specimen shows an opposite modification of the groundplan, in that there is lack of expression of the terminal fascia at the costa, making this interfascial area appear larger (Fig 6E).

Overall, *Micropterix rothenbachii* is the most variable of the 12 species examined. Of the 20 wings examined, 15 have six differentiated fasciae and no color fields (Figs 5I and 6D). One wing is unique in having no apparent terminal fascia due to lack of expression (Fig 6E). Two wings have color fields formed by suffusion of the interfascial area between the preterminal + terminal fasciae (Fig 5I). Two wings have only two differentiated fasciae, basal and subbasal, and two color fields formed by confluence of the median + postmedian and preterminal + terminal fasciae (Fig 5I).

## Discussion

Because very few forks in the wing veins align with the edges of fasciae, *Micropterix* shows little support for Lemche’s “vein-fork” model. Forewing patterns in *Micropterix* fit the “wing-margin” model. The five fasciae known from tortricids occur along the wing costa exactly as predicted by the model. On the wing costa in both Tortricidae and *Micropterix*, the basal and subbasal fasciae occur basal to the Sc vein (Sc_2_ in *Micropterix*), the median fascia straddles the Sc vein, the postmedian fascia straddles R_1_ (R_1b_ in *Micropterix*), and the preterminal fascia straddles Rs_2_. A terminal fascia abuts or straddles Rs_4_ in *Micropterix*, but this fascia was not defined for Tortricidae in the "wing margin" model because this vein terminates on the outer margin (termen), not the costa, of tortricid wings (Fig 1B). Rather, the terminal fascia in *Micropterix* may correspond with what is known in some species of Tortricidae as an “apical spot.” Regardless, when present in *Micropterix*, the terminal fascia follows the pattern that was first recognized in the five tortricid fasciae: among the Rs veins, each fascia straddles/abuts one vein at the costa, and all fasciae are separated by an interfascial area that also straddles/abuts one vein at the costa. The *Micropterix* groundplan requires a slight alteration of the “wing-margin” model due to differences in wing shape between Micropterigidae and Tortricidae—in both families, the underlying concept is the same: at the costa, each fascia and each interfascial area straddles or abuts one vein; beyond R_1_, all primitive veins are visible in both Micropterigidae and Tortricidae and the venation-fascia relationship can be readily observed.

None of the instances of incomplete suffusion or lack of expression at the costa violate the “wing-margin” model. When an interfascial area is incompletely suffused with dark color, causing an adjacent fascia to appear larger, this seemingly enlarged fascia continues to straddle the vein originally predicted by the model. When there is lack of expression of a fascia, causing an adjacent interfascial area to appear larger, the interfascial area continues to straddle the vein originally predicted by the model.

In recent years, representatives of *Micropterix* have been included in a number of molecular phylogenies [38,51,52], including one study devoted strictly to this genus [53]. However, the most complete phylogeny of *Micropterix* is limited to 27 of the 77+ described species in the genus. Ten of the 12 species examined here are included in this phylogeny and are dispersed throughout the clade. For this reason, it is not yet possible to determine the directionality of wing pattern evolution (e.g., whether color fields or differentiated fasciae are the primitive condition).

In the original “wing-margin” model proposed for Tortricidae (Fig 1B), fasciae straddle alternating veins on both the costal and inner margins of the wing. In *Micropterix*, the wing pattern groundplan is not nearly as clear on the inner margin (dorsum) as it is on the costal margin (costa) due to extensive suffusion of interfascial areas. The evidence available from *Micropterix* suggests no firm conclusions about the relationship between fasciae and the inner margin.

Developmentally, this groundplan requires a mechanism through which vein position could constrain pattern elements even when veins are not expressed in the adult wing. This phenomenon has been observed in other Lepidoptera—for example, the “Cu2” and “Pc” eyespots on nymphalid wings are separated by a vein that is not maintained in the adult wing [54]–though the details are still poorly understood. Studies of wing vein development in microlepidoptera are lacking. In butterflies, the transcription factors Notch and Distal-less serve as markers during wing vein development [55] and the transcription factors Spalt, Cubitus interruptus, and Engrailed are sector- or compartment-specific [2,56]; transcription factors of this sort could produce the *Micropterix* groundplan.

The relationship between wing pattern and venation in *Micropterix* and Tortricidae allows for a nomenclatural system that is not merely positional, but instead is based on the underlying skeletal character of venation. Previously, descriptions of wing pattern in microlepidoptera have used a variety of nomenclatural systems that are based simply on whether a given wing pattern element occurs proximally or distally on the wing. Under such a system, the fasciae that straddle Rs_2_ in Tortricidae (Fig 1B) and Rs_4_ in *Micropterix* (Fig 5A) could be described with identical nomenclature because both fasciae would be the most distally positioned. However, under the nomenclatural system used here—first laid out for Tortricidae in the “wing-margin” model, and now applied to *Micropterix*–terminology used for fasciae is based on venation, a skeletal character and not merely position along the wing.

**Fig 7:**
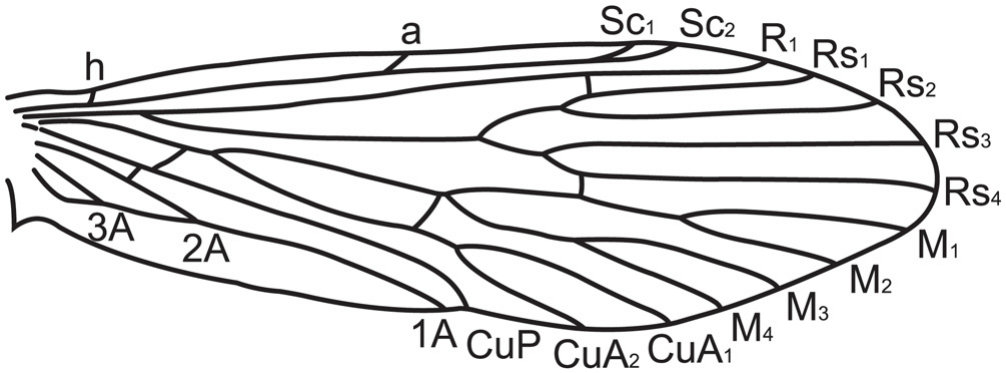
The wing venation of *Rhyacophila fuscula* (Trichoptera). From *The Wings of Insects* [61].

**Fig 8:**
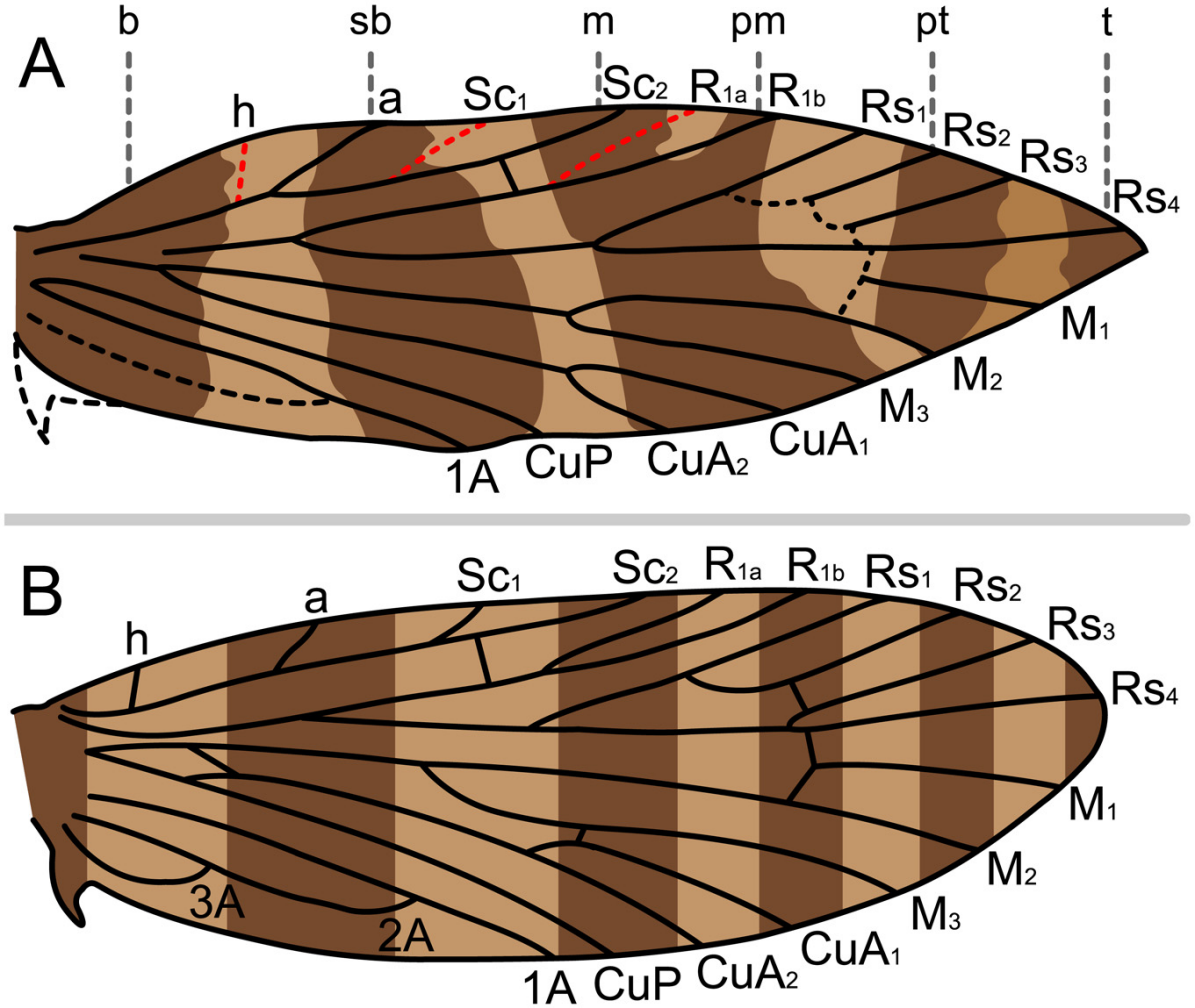
Predicted relationships between wing patterning and venation in basal Lepidoptera. (A) The wing pattern groundplan of *M*. *schaefferi* 2, showing the possible distribution of primitive veins that are not visible in *Micropterix*. (B) A hypothesized primitive wing pattern groundplan for Lepidoptera. Based on the most recent hypothesis for primitive wing venation [46].

In *Micropterix*, as in Tortricidae, not all boundaries between fasciae and interfascial areas (marked by strigulae on tortricid wings) are separated by veins—three additional veins would be needed in order for each fascia to straddle one vein and for all fasciae to be separated by one vein. This is likely due to ancestral veins that are not expressed in the adult stages of these taxa. Between veins R_1b_ and Rs_4_, all of which are present in *Micropterix*, fasciae and interfascial areas all straddle or abut one vein. Between the base of the wing and the R_1b_ vein, veins are known to be missing; *Micropterix* has only two veins in this area of the wing, but other micropterigid genera, as well as extinct basal Lepidoptera, have four: h, Sc_1_, Sc_2_, and R_1a_ [46,57–59]. If the two additional, ancestral veins—h and Sc_1_ –are included in the *Micropterix* wing pattern groundplan, only one more vein is needed in order for all fasciae to straddle one vein and to be separated by one vein. Such a vein—located basally to R_1_ along the costa, and not expressed in the wings of adult Lepidoptera—is a plesiomorphic mecopteroid feature known from many fossil Amphiesmenoptera that lived during the Permian Period, before Trichoptera and Lepidoptera diverged [60], and can be found between the h and Sc_1_ veins on the forewings of some extant basal Trichoptera, e.g. *Rhyacophila fuscula* (Fig 7) [61]. Comstock termed this vein “a” in *Rhyacophila* [61]. This vein occurs in very few species of Trichoptera and, perhaps for this reason, is not mentioned in recent treatments of the trichopteran wing groundplan [62], but its presence was confirmed by our own examinations of *Rhyacophila fuscula* in the Mississippi Entomological Museum. When plotted in order along the costa of the *M*. *schaefferi* 2 forewing, the aforementioned veins produce a groundplan in which each fascia-interfascial boundary is separated by one vein (Fig 8A). This suggests a new groundplan for primitive wing patterning in ancestral moths (Fig 8B).

This groundplan has implications for wing vein homology in Lepidoptera. Of the two visible veins that precede R_1b_ in *Micropterix*, one is straddled by the subbasal fascia at the costa and the other is straddled by the median fascia. According to the “wing-margin” model, these veins must be “a” and Sc_2_. However, in basal moths such Micropterigidae and ancestral Lepidoptera, these veins have long been referred to as Sc_1_ and Sc_2_ [46,47,61,63,64]. Our analysis of wing pattern suggests that, particularly in taxa that are closely related to *Micropterix*, the vein that is often termed Sc_1_ may in fact be Comstock’s trichopteran “a.”

## Conclusions

Along the costa, fasciae always occur between the same wing veins regardless of how many instances of suffusion or lack of expression have occurred. The fascia-venation relationship is the same in *Micropterix* as in Tortricidae despite the many millions of years of evolutionary history that separate these two lineages. The similar wing pattern groundplans in Micropterigidae and Tortricidae suggest that fasciae, not spots, are the primitive wing pattern elements for Lepidoptera. The results reported here also suggest that these fasciae are homologous between the families Micropterigidae and Tortricidae, which would strongly imply that these wing pattern elements are primitive in Lepidoptera and homologous in all taxa in which they are present. Future research should focus on other genera within the Micropterigidae, and on the many superfamilies of Lepidoptera that bridge the phylogenetic gap between Micropterigidae and Tortricidae, in order to determine the prevalence of fasciate wing patterns that fit the “wing-margin” model.
